# Autonomic Nervous System Function and Sensory Sensitivity in 12-Month-Old Infants with the *FMR1* Premutation

**DOI:** 10.3390/ijms27156542

**Published:** 2026-07-23

**Authors:** Amelia Morton, Holley Arnold, Lisa Hamrick, Abigail Chase, Stacey Cobb, Jane Roberts

**Affiliations:** 1 Floyd School of Medicine, University of South Carolina, Columbia, SC 29209, USA; amelia.morton@uscmed.sc.edu (A.M.); abigail.chase@uscmed.sc.edu (A.C.); stacey.cobb@prismahealth.org (S.C.); 2 Department of Psychology, University of South Carolina, Columbia, SC 29208, USA; holley.pitts.arnold@sc.edu (H.A.); rague@mailbox.sc.edu (L.H.); 3 Carolina Autism and Neurodevelopment Research Center, University of South Carolina, Columbia, SC 29208, USA; 4 Prisma Health Children’s Hospital, 7 Richland Medical Park Dr, Columbia, SC 29203, USA

**Keywords:** *FMR1* premutation, CGG repeat length, autonomic nervous system, respiratory sinus arrhythmia, interbeat interval, hyporesponsive, hyperresponsive

## Abstract

The fragile X premutation (FXpm) is a relatively common condition caused by an expansion of 55–200 cytosine–guanine–guanine (CGG) repeats in the fragile X messenger ribonucleoprotein 1 (*FMR1)* gene. Previous studies have identified sensory processing challenges in children with the FXpm and reduced autonomic regulation in FXpm infants; no research has examined the relationship between autonomic nervous system (ANS) functioning or molecular variables and sensory responsiveness during infancy. This study examined parent-reported sensory responsiveness and its association with baseline respiratory sinus arrhythmia (RSA), interbeat interval (IBI), and CGG repeat length in 12-month-old infants with the FXpm (*n* = 35) and neurotypical (NT) controls (*n* = 55). Results indicated no significant differences in hyporesponsive or hyperresponsive sensory behaviors and no significant associations between baseline RSA or IBI and sensory responsiveness in either group. Within the FXpm group, however, greater CGG repeat length was associated with lower hyperresponsive sensory scores. These findings suggest that sensory processing differences may not be behaviorally evident at 12 months of age despite the presence of biological variability associated with the premutation. The study contributes to the emerging literature on early FXpm development and highlights the importance of examining genetic and physiological factors that may precede later-emerging behavioral phenotypes.

## 1. Introduction

Fragile X messenger ribonucleoprotein 1 (*FMR1*) associated conditions arise by a gene mutation in the *FMR1* gene located on the X chromosome. This mutation results in abnormal expansion of cytosine–guanine–guanine (CGG) repeats in the 5’UTR portion of the gene. *FMR1*-associated conditions are categorized based on the expansion of CGG repeats as either fragile X syndrome (FXS) or fragile X premutation (FXpm). FXS, also commonly referred to as the full mutation, is diagnosed when an individual has greater than 200 CGG repeats, whereas individuals with between 55 and 200 CGG repeats are classified as having the premutation (FXpm). This abnormal trinucleotide expansion results in a deficiency in the transcription of *FMR1* protein (FMRP), a protein known to play a significant role in healthy brain development, neuronal synaptogenesis and plasticity, and regulating mRNA translation [1]. FXS is a rare syndrome, while the FXpm is a common condition with an estimated prevalence of 1:290–855 in males and 1:148–300 in females [2,3,4].

Previously, individuals with the FXpm were thought to be unaffected carriers with no clinical deficits other than the risk of transmission and expansion of the mutation to their offspring. However, extensive literature over the past two decades has unambiguously demonstrated that there is a subgroup of individuals with the FXpm who experience a range of impairment across cognitive, affective, and health-related domains. These include fragile-X-associated tremor/ataxia syndrome [5,6,7], fragile X primary ovarian insufficiency syndrome [8], and neuropsychiatric disorders [9,10]. These conditions are associated with both environmental factors like educational attainment or social support as well as molecular factors including CGG repeat length, reduced FMRP, and elevated mRNA [10,11].

Despite the growing literature on the FXpm, clinical knowledge regarding the development of infants and children with the premutation remains extremely limited. However, emerging research has linked the FXpm to a range of cognitive and behavioral phenotypes, including autism spectrum disorder (ASD), anxiety, depression, attention-deficit/hyperactivity disorder (ADHD), seizure disorders, and social communication impairments [12,13,14,15,16]. Because many of these findings are based on relatively small or clinically referred samples, studies of non-clinically referred infants are needed to better characterize the early developmental phenotype and identify factors associated with later neurodevelopmental outcomes. Neuropsychiatric conditions associated with the FXpm have been referred to as fragile-X-associated neuropsychiatric disorders (FXAND) [10]. While most children with the FXpm have average cognitive abilities, some display developmental delays or elevated risk for co-occurring conditions [15,16]. ASD may be one of the most common FXAND conditions in children with the FXpm, with up to 79% of clinically referred individuals meeting diagnostic criteria [17]. However, there is a wide range of prevalence estimates largely driven by recruitment methods with a range of 14–19% diagnosed with ASD in non-clinically referred FXpm samples [12]. This rate is substantially higher than the rate of 3.2% in the U.S., signaling the importance of research looking at ASD and related features [18]. Social communication is a core feature of ASD and impairments in social communication appear elevated in children with the FXpm [14,16].

While the FXpm neuropsychiatric phenotype in children is of increasing research focus, significant gaps remain. One important research gap is understanding the early predictors of sensory processing, given the important role sensory processing plays across cognitive, social, and affective domains. Sensory processing is a broad term that includes hyporesponsiveness and hyperresponsiveness. Hyporesponsiveness is defined as a lack of a response or a reduced intensity in the reaction to a sensory stimulus; for example, not reacting to a loud noise or physical touch meant to get attention [19]. Hyperresponsiveness refers to an exaggerated reaction or response to a sensory stimulus [20]. This response may involve covering ears in response to a slight change in volume or reactions that are much faster or intense than expected with typical sensory responsiveness. Sensory processing differences are increasingly conceptualized as a transdiagnostic dimension of neurodevelopmental psychopathology rather than a disorder-specific characteristic. Sensory hyperresponsiveness and hyporesponsiveness are observed across ASD, FXS, ADHD, anxiety disorders, and other neurodevelopmental conditions, suggesting the involvement of shared underlying neurobiological mechanisms rather than diagnosis-specific processes [21,22,23]. Within *FMR1*-associated conditions, sensory processing abnormalities are well documented in children with FXS [24]. Emerging evidence suggests sensory processing disruptions in infants or young children with the FXpm, which supports the hypothesis that atypical sensory responsivity may represent an early developmental marker of broader neurobehavioral vulnerability rather than a phenotype unique to a specific diagnostic outcome [25,26]. This transdiagnostic framework is particularly relevant to the study of infants with the FXpm, as early sensory differences may precede and potentially contribute to later-emerging neurodevelopmental and neuropsychiatric outcomes including ASD, anxiety, and other manifestations encompassed within FXAND [10].

To date, two preliminary studies have examined sensory processing in infants or very young children with the FXpm. Raspa and colleagues [26] collected parental reports via an online survey that included children 5–18 years of age across three groups: FXpm (*n* = 14), FXS (*n* = 129), and neurotypical (NT) comparisons (*n* = 33). Using the Brain Body Center Sensory Scales, they reported that children with the FXpm exhibited greater sensory sensitivity across all subscales compared to NT controls, with levels slightly lower than those observed within FXS [26]. The FXpm sample displayed sensory atypicalities on 90% of the items with hypersensitivity as one of the highest rated sensory challenges. Their findings support the conclusion that a subset of individuals with the FXpm shares a clinical phenotype with individuals with FXS, though the phenotype is generally less severe. These results are consistent with an exploratory study [16] that reported a small sample of infants (*n* = 18) and toddlers with the FXpm demonstrated consistent hyporesponsiveness from 6 to 30 months of age using the Sensory Experience Questionnaire (SEQ). Hyperresponsivity was also present with an increase in severity across age, which contrasted with the decrease in severity observed in NT children [16]. Results from this study indicated that ~25% of the scores from 12-month-old infants fell in the deficient range. Together, these two preliminary studies suggest that sensory processing differences may be present during early development in children with the FXpm. However, both studies included small samples, and only one included children younger than 5 years of age, highlighting the need for additional research in larger infant cohorts.

Several mechanisms have been theorized to explain sensory sensitivity in the FXpm population, including underlying molecular factors such as lower levels of FMRP, faulty GABA inhibition, mitochondrial dysfunction, and CGG repeat length [12,27,28]. While some studies suggest a linear relationship between CGG repeat expansion and symptom severity, others report a curvilinear pattern, particularly in females, with mid-range repeats associated with greater impairment [29,30,31]. In research with young children with the FXpm, evidence is mixed with one study demonstrating a linear relationship between longer CGG repeat length and lower developmental scores in preschoolers [16] while another study demonstrated no relationship between CGG repeat length and autonomic function in infants with the FXpm [32]. No research has examined the relationship of CGG repeat length to sensory processing for infants with the FXpm to date.

Sex differences may also contribute to sensory processing profiles in the FXpm as some evidence suggests that FXpm males exhibit greater sensory sensitivity than FXpm females [26]. However, there were no sex effects observed in sensory processing patterns in infants with the FXpm [16]. Sex differences in the FXpm are likely attributable to the X-linked nature of the *FMR1* gene. Females possess two X chromosomes and typically carry one normal and one premutation *FMR1* allele. Random X-chromosome inactivation leads to variability in the expression of the normal and premutation alleles across cells, contributing to the more heterogeneous and generally less severe clinical phenotype observed in females compared to males with the FXpm [33,34].

Determining the age at which sensory processing difficulties emerge in individuals with the FXpm is important because these differences may serve as early indicators of neurodevelopmental vulnerability, provide insight into the developmental trajectory of the premutation phenotype, and inform the timing of screening and intervention efforts. Evidence suggests that sensory impairments in individuals with the FXpm emerge early in development but follow distinct trajectories, with hyporesponsivity detectable by 6 months of age and hyperresponsivity emerging during the second or third year of life [16]. Despite variability in the timing of onset, sensory sensitivities appear to be elevated and relatively stable by middle childhood, as no differences have been observed between children aged 12 years and younger and those older than 12 years [26].

One mechanism that has not yet been examined in FXpm research is the role of the autonomic nervous system (ANS) in sensory processing. The ANS plays a critical role in sensory processing by regulating physiological arousal and modulating responses to environmental stimuli. Dysregulation of sympathetic and parasympathetic activity may contribute to atypical sensory experiences, including hyperresponsivity, hyporesponsivity, and difficulties with sensory regulation, and has been implicated in the sensory phenotypes observed in FXS and the FXpm [24,35,36]. Cardiac measures provide a non-invasive method for assessing ANS function in real time. Interbeat interval (IBI) is defined as the time between consecutive heartbeats, which reflects general physiological arousal and is influenced by both parasympathetic and sympathetic activity. In contrast, respiratory sinus arrhythmia (RSA) is the cardiac indicator that isolates the parasympathetic and vagal regulation [37,38]. RSA is the measurement that represents heart rate variability (HRV) or the variability in the IBI measurements corresponding with spontaneous breathing [38].

The Polyvagal and Neurovisceral Integration Theories provide a useful framework for understanding the relationship between ANS function and sensory processing [39,40,41]. These theories posit that a dysfunctional ANS system could contribute to sensory processing impairments in the FXpm. Consistent with this framework, reduced baseline RSA has been associated with pragmatic language deficits in FXpm adults, indicating that vagal regulation may contribute to broader phenotypic expression [29]. In the only study to date that has examined ANS function in infants with the FXpm, Chase and colleagues [32] reported lower baseline RSA and no difference in IBI in 12-month-old infants with the FXpm compared to age-matched NT controls. This was consistent with previous findings associated with reduced RSA in FXS children, including those with and without ASD and anxiety phenotypes [36,42,43]. Studying ANS function in infants with the FXpm may provide critical insight into the biological mechanisms underlying early sensory processing differences. Because the ANS regulates physiological arousal and responses to environmental stimuli, atypical autonomic functioning may contribute to the emergence of sensory hyperresponsivity and hyporesponsivity observed in fragile-X-related conditions. Characterizing ANS function during infancy may therefore help identify early markers of neurodevelopmental vulnerability, clarify pathways linking the premutation to sensory outcomes, and inform efforts toward early identification and intervention. Despite the clear rationale for examining ANS in young children with the FXpm, there has been no examination of the relationship between ANS functioning and sensory processing in FXpm infants. Most existing studies have focused on individuals with FXS, adult FXpm women, or older children with the FXpm, leaving a critical gap in early development.

The present study addresses multiple gaps in the pediatric FXpm literature by characterizing sensory processing patterns in a relatively large and unbiased sample of 12-month-old infants with the FXpm contrasted to NT controls and examining whether ANS or genetic markers are related to sensory processing in the infants with the FXpm. We hypothesize that the infants with the FXpm will display elevated sensory processing impairments that are related to reduced RSA, shorter IBI, and longer CGG repeat length.

This study addresses two primary research questions:

1. Does parent-reported sensory processing differ in 12-month-old infants with the FXpm contrasted to NT controls?
2. Are baseline cardiac measures (IBI and RSA) or CGG repeat length related to parent-reported sensory processing in 12-month-old infants with the FXpm?

## 2. Results

### 2.1. Preliminary Analyses

Analyses indicated that the FXpm and NT groups did not significantly differ on chronological age (*t*(88) = 0.144, *p* = 0.886, *d* = 0.031). Given reported sex effects and a greater proportion of males in the NT group compared to the FXpm group (Table 1), we examined potential sex effects. Preliminary analyses indicated that males and females did not significantly differ in SEQ hyporesponsive scores (*t*(88) = 0.204, *p* = 0.839, *d* = 0.046), SEQ hyperresponsive scores (*t*(88) = 0.370, *p* = 0.712, *d* = 0.083), baseline IBI (*t*(88) = 1.250, *p* = 0.215, *d* = 0.280), or baseline RSA (*t*(88) = 0.818, *p* = 0.415, *d* = 0.183). Thus, we report analyses using the full sample of males and females in each group to maintain the highest level of power.

### 2.2. Sensory Processing in the FXpm Contrasted to NT Controls

**Figure 1:**
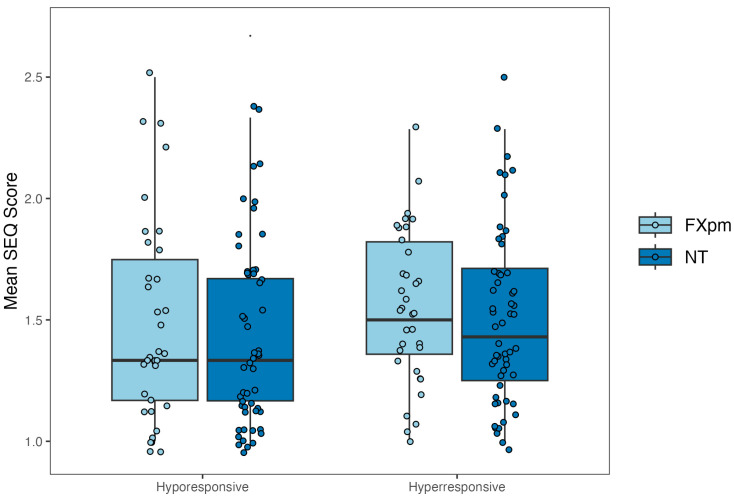
Hyporesponsive and hyperresponsive SEQ scores by group. Note: groups did not differ significantly in SEQ Hypo- or Hyperresponsiveness scores. FXpm = fragile X premutation, NT = neurotypical, SEQ = Sensory Experiences Questionnaire.

As shown in Figure 1, the FXpm and NT groups did not differ in SEQ hyporesponsive scores (*t*(88) = 0.409, *p* = 0.684, *d* = 0.088) or hyperresponsive scores (*t*(88) = 0.826, *p* = 0.411, *d* = 0.179), with small effect sizes observed. Although the FXpm group had a somewhat higher proportion of children in the at-risk/deficient category than the NT group (22.9% vs. 16.4%), this difference is not significant (*χ*^2^ = 0.589, *p* = 0.443).

### 2.3. Association of ANS with Sensory Scores Across Diagnostic Groups

**Figure 2:**
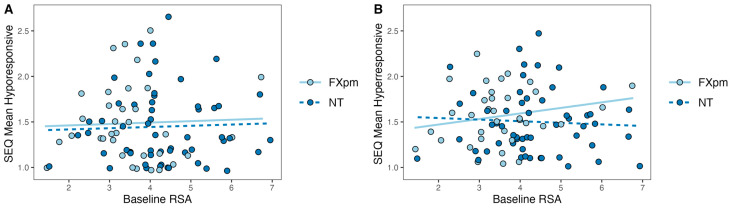
Hypo- and hyperresponsive by RSA for both FXpm and NT infants. Note: distribution of (**A**) Hyporesponsive and (**B**) Hyperresponsive in FXpm and NT groups. There were no significant associations between RSA and SEQ Hypo- or Hyperresponsiveness. FXpm = fragile X premutation, NT = neurotypical, RSA = respiratory sinus arrhythmia, SEQ = Sensory Experiences Questionnaire.

**Figure 3:**
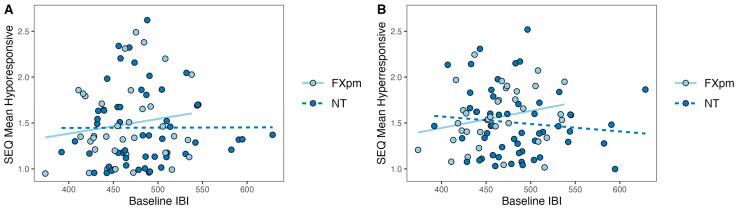
Hypo- and hyperresponsive by IBI for both FXpm and NT infants. Note: distribution of (**A**) Hyporesponsive and (**B**) Hyperresponsive in FXpm and NT groups. There were no significant associations between IBI and SEQ Hypo- or Hyperresponsiveness. FXpm = fragile X premutation, IBI = interbeat interval, NT = neurotypical, SEQ = Sensory Experiences Questionnaire.

Correlation analyses were performed for baseline RSA (Figure 2) and baseline IBI (Figure 3) with hypo- and hyperresponsive SEQ scores for both FXpm and NT infants. As depicted, there was no significant association between baseline RSA and hyporesponsive SEQ scores in either FXpm infants (*r* = 0.039; *p* = 0.822) or NT infants (*r* = 0.037; *p* = 0.788). No statistically significant correlation was found between baseline RSA and hyperresponsive SEQ scores in either group as well (FXpm: *r =* 0.206, *p* = 0.236; NT: *r* = −0.055, *p* = 0.689). Additionally, there was not a significant relationship between baseline IBI and hyporesponsive SEQ scores in the FXpm (*r* = 0.154; *p* = 0.376) or NT (*r* = 0.003; *p* = 0.981) groups, nor a statistical correlation with baseline IBI values and hyperresponsive scores (FXpm: *r* = 0.241, *p* = 0.163; NT: *r* = −0.108, *p* = 0.430).

### 2.4. Association of CGG Repeats with Sensory Scores in FXpm Infants

**Figure 4:**
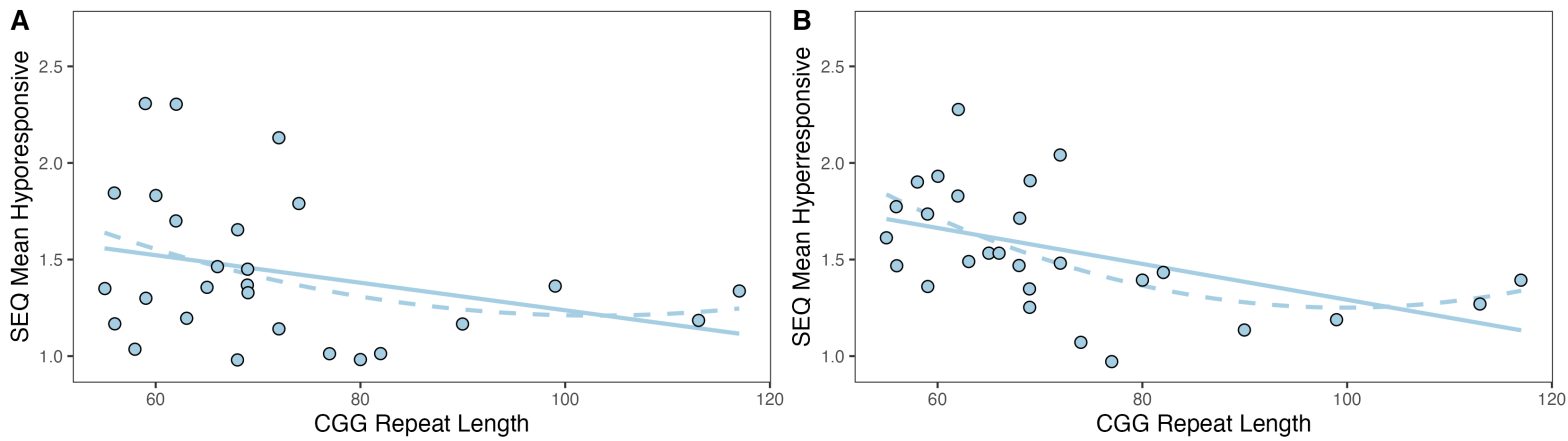
Hypo- and hyperresponsive by CGG Repeat Length in FXpm infants. Note: CGG repeat length as a function of (**A**) Hyporesponsive and (**B**) Hyperresponsive in FXpm. The overall model predicting the linear and quadratic effects of CGG repeats on SEQ hyperresponsive scores was statistically significant, with fixed effects in the hyperresponsive sensory scores demonstrating a marginal statistical effect. CGG = Cytosine–guanine–guanine, SEQ = Sensory Experiences Questionnaire.

Figure 4 depicts the relationship between CGG repeat length and hypo- and hyperresponsive SEQ scores in the FXpm group. The overall linear and quadratic effects of CGG repeats on SEQ hyporesponsive scores in infants with the FXpm were not statistically significant (*R*^2^ = 0.104, *F*(2, 24) = 1.397, *p* = 0.267). The linear effect of CGG repeats did not significantly predict SEQ hyporesponsive scores (*β* = −0.038, *p* = 0.384), nor did the quadratic effect (*β* = 0.000, *p* = 0.475). The overall linear and quadratic effects of CGG repeats on SEQ hyperresponsive scores in infants with the FXpm were statistically significant (*R*^2^ = 0.557, *F*(2, 24) = 5.395, *p* = 0.012). The linear effect of CGG repeats marginally predicted SEQ hyperresponsive scores (*β* = −0.058, *p* = 0.062), such that one CGG repeat would result in a 0.058-point decrease in SEQ hyperresponsive score. The quadratic effect of CGG repeats was not a statistically significant predictor of the SEQ hyperresponsive scores in FXpm infants (*β* = 0.000, *p* = 0.111).

## 3. Discussion

The FXpm is a common genetic variant associated with an elevated likelihood of cognitive, psychological, and health-related challenges throughout one’s lifespan [10,29,44]. Despite its high prevalence and risk for impairment, most research on the FXpm focuses on adults, with only a handful of studies focused on early development with a non-clinically referred sample. The present study examined sensory responsiveness and its association with ANS functioning and genetic factors in 12-month-old infants with the FXpm compared to NT infants. Results indicated no significant group differences in hypo- or hyperresponsive sensory behaviors, and no associations between baseline ANS measures (RSA, IBI) and sensory responsiveness in either group. However, within the FXpm group, CGG repeat length demonstrated a marginal association with hyperresponsive sensory scores, suggesting that genetic variation may relate to early sensory phenotypes even in the absence of observable group-level differences.

The absence of group differences in sensory responsiveness between FXpm and NT infants is consistent with the possibility that behavioral manifestations of sensory sensitivity may not yet be evident at 12 months of age, although additional methodological and developmental factors may also contribute to these findings. It is possible that subtle sensory differences in infancy are more difficult to detect using caregiver-report measures alone. Future studies integrating caregiver reports with laboratory-based observational paradigms and physiological measures may provide a more comprehensive characterization of early sensory phenotypes. Our findings critically extend the only existing study to examine sensory processing in a small sample of infants with the FXpm (*n* = 18). Like Wheeler and colleagues [16], we found a similar subset of infants with SEQ total scores in the deficient/at risk range (22.9% vs. ~25%, respectively), no group-level differences in hyperresponsivity at 12 months, and no sex effects. However, Wheeler reported elevated hyporesponsiveness in 12-month-olds with the FXpm while we did not replicate this finding in our larger sample. These differences could be due to the longitudinal approach in the Wheeler study as their results clearly showed increased hyporesponsivity across age, whereas our study is cross-sectional. Alternatively, Wheeler’s findings could have been spurious based on the smaller sample size. Longitudinal follow-up in a large sample is essential to address these questions.

Our findings align with the broader characterization of the FXpm as a condition in which phenotypic features are often subtle and not clinically apparent in early life. From a developmental perspective, infancy may represent a preclinical stage during which underlying biological differences have not yet been translated into observable behavioral phenotypes. Indeed, longitudinal evidence supports this interpretation as Wheeler and colleagues [16] reported that infants with the FXpm exhibited increasing divergence from NT peers over time, with differences in sensory responsiveness becoming more pronounced after 12 months of age. Additionally, features associated with FXAND typically emerge later in development (e.g., anxiety, ASD) and persist into adulthood [10,32,44,45]. As such, the current findings may reflect the developmental lag between genetic risk and its behavioral expression, rather than the true absence of meaningful group differences.

Consistent with the absence of group differences in sensory responsiveness, no significant associations were observed between baseline RSA and IBI and sensory responsiveness in either group. These findings were surprising given prior evidence linking autonomic regulation to sensory processing across development in NT infants and those with ASD [46,47,48]. Notably, Chase et al. [32] reported lower baseline RSA in infants with the FXpm compared to NT controls, while Wheeler et al. [16] identified increased sensory seeking and hyporesponsivity in FXpm infants across early development. Together, these findings informed the hypothesis that reduced baseline RSA would be associated with increased hyporesponsivity in FXpm infants. The lack of association observed in this study suggests that resting-state autonomic regulation alone may not account for early sensory phenotypic expression in the FXpm population. Alternatively, the relationship between autonomic regulation and sensory responsiveness may be more apparent under dynamic or experimentally induced conditions than during resting-state. These findings also have implications for theoretical models linking autonomic regulation to sensory processing, such as polyvagal and regulatory frameworks, which propose that physiological state influences behavioral responsiveness to environmental stimuli [39,48,49]. The absence of such associations at 12 months of age suggests that these relationships may not be fully established in early infancy or may require more dynamic contexts to be detected. Thus, the current findings refine theoretical models by emphasizing the importance of developmental timing and measurement context. These results point to the likelihood of additional mechanisms, such as central neural processing, genetic variability, and context-dependent physiological reactivity (e.g., baseline vs. during a sensory processing experiment). Future studies should therefore examine ANS functioning during dynamic sensory experiences to better characterize the role of physiological regulation in emerging sensory phenotypes rather than a reported objective measure of sensory responsiveness.

While group comparisons and ANS measures did not reveal significant associations with sensory responsiveness, analyses within the FXpm group highlighted the potential importance of genetic variability. Our study identified a significant inverse association between CGG repeat length and hyperresponsive sensory scores in infants with the FXpm, with evidence of linear, and potentially, curvilinear effects. Greater CGG repeat length was associated with lower hyperresponsive scores. Prior research examining CGG repeat length has largely focused on adult FXpm populations, with findings supporting both linear and curvilinear relationships between repeat length and phenotypic outcomes [16,29,30,31,50]. For example, Wheeler [16] reported that increased CGG repeat length was associated with poorer developmental outcomes in infants with the FXpm, whereas studies in adult women have demonstrated curvilinear effects, with individuals in the mid-range of the premutation showing greater impairment [29,30,31]. Research examining CGG repeat length in relation to early sensory phenotypes has been limited and often underpowered, yielding largely non-significant findings. Additionally, prior work has shown that CGG repeat length does not predict baseline ANS measures in 12-month-old FXpm infants [32]. The present findings provide preliminary evidence that genetic variation within the premutation range may contribute to variability in early sensory responsiveness. Although CGG repeat length alone is unlikely to fully explain phenotypic outcomes, the observed association suggests that genetic factors may influence sensory development in infancy. The inverse association observed in the present study also underscores the complexity of genotype–phenotype relationships in the *FMR1* premutation. Prior work has demonstrated that phenotypic outcomes are not always linearly related to CGG repeat length, suggesting that multiple molecular mechanisms, including changes in *FMR1* mRNA expression, FMRP production, and RNA toxicity, may interact to influence developmental outcomes. Future longitudinal studies incorporating molecular biomarkers alongside behavioral measures will be important for clarifying the biological mechanisms underlying early sensory development and determining the stability and developmental significance of this relationship. Given the modest sample size, these findings should be considered exploratory and require replication in larger longitudinal cohorts to clarify the stability and developmental significance of this relationship.

Together, these findings provide preliminary evidence that biological variability may precede observable behavioral differences in infants with the *FMR1* premutation. Although these findings require replication in larger longitudinal cohorts, they support the importance of integrating genetic, physiological, and behavioral measures when characterizing early phenotypic development. Such multimodal approaches may improve the identification of infants at increased likelihood for later neurodevelopmental challenges.

Several limitations should be considered when interpreting these findings. Although this study includes the largest non-clinically referred sample of 12-month-old infants with the FXpm to date to our knowledge [16,26], the overall sample size remains modest, particularly for analyses involving CGG repeat length, which were restricted to a subsample with available data. This may have limited statistical power to detect group differences or more nuanced associations. This is particularly relevant for our analyses that explore the effects of CGG repeat length on hypo- and hyperresponsiveness, as only a subset of our full sample had available CGG repeat data. Thus, null findings related to this research question should be interpreted with caution and explored in larger, more highly powered samples. Although no sex differences were observed, the sample may not have been sufficiently powered to detect sex-specific effects. Additionally, the cross-sectional design precludes conclusions regarding developmental trajectories and limits our ability to determine how ANS functioning and sensory responsiveness evolve over time. ANS functioning was assessed using baseline measures only, which may not capture the context-dependent or reactive physiological processes that are deeply relevant to sensory experiences. Furthermore, sensory responsiveness was measured via parent report, which may introduce reporting bias and reduce measurement precision as subtle sensory differences during infancy may be more difficult for caregivers to observe consistently than behaviors that emerge later in development. Future research would benefit from larger, longitudinal samples and the inclusion of experimentally induced or ecologically valid sensory paradigms to better characterize the dynamic interplay between physiological regulation and sensory development in the FXpm.

The present findings contribute to the emerging literature on early development in infants with the *FMR1* premutation by demonstrating that parent-reported behavioral differences in sensory responsiveness were not evident at 12 months of age, despite preliminary evidence that genetic variation may contribute to variability in early sensory responsiveness. These findings highlight the importance of continued longitudinal investigation to determine how early biological markers relate to later-emerging neurodevelopmental outcomes. Future studies integrating molecular, physiological, and behavioral measures across development will be important for clarifying the developmental trajectory of the *FMR1* premutation.

## 4. Materials and Methods

### 4.1. Participants

This cross-sectional study included 90 infants (FXpm: *n* = 35; NT: *n* = 55), mean ages of 11.34 and 11.29 months, respectively, drawn from longitudinal studies of early development of *FMR1* conditions (R01MH090194; R01HD106652). Inclusion criteria were consistent across groups and required full-term birth and normal hearing and vision. FXpm participants were recruited through the Institute for Basic Research Specialty Clinical Laboratories (IBR SCL), social media, and through referrals from FXS research collaborators. The IBR SCL collected and analyzed prenatal samples with the collaboration of genetic counselors who informed eligible families about this study. Because FXpm participants were not recruited through clinical referrals, this sample is less biased toward cases that are associated with severe clinical presentations, making it more representative of the general FXpm population. All participants had the FXpm confirmed through genetic analyses; however, only a subset (27 out of 35) had reports that included the number of CGG repeats.

NT children were recruited from the community and were excluded if there was a family history of FXS, ASD, or a related diagnosis. NT participants were also excluded if they demonstrated significant developmental delays (i.e., >2 standard deviations below the mean in any developmental domain) on either the Bayley Scales of Infant and Toddler Development, Fourth Edition (BSID) [51] or Mullen Scales of Early Learning (MSEL) [52] developmental assessments for their selected assessment. Given that some children in the NT group have not yet reached an age where clinical outcomes can be assessed, it is possible that some children in the NT group may later demonstrate a non-typically developing outcome at 24 months.

For participants who had multiple assessments, the assessment closest to 12 months of age was prioritized to maximize the amount of data available while still retaining a focus on the early infant developmental period when phenotypic differences in the FXpm have been found to emerge [16]. All infants with baseline heart activity data (FXpm: *n* = 35; NT: *n =* 55) and CGG repeat number (FXpm: *n* = 27; 15 males) were included for those respective analyses.

### 4.2. ANS Function

Baseline autonomic nervous system function was derived from electrocardiogram (ECG) data collected using a telemetry-based monitor [53,54] sampling at 1024 Hz. Infants wore a two-electrode heart rate monitor while watching a 3 min silent Baby Einstein video containing music and sound effects. Interbeat interval (IBI) data were visually inspected and edited for artifacts and arrhythmia using CardioEdit Plus V6 (Brain–Body Center for Psychophysiology and Bionegineering, University of North Carolina, Chapel Hill, NC, USA) [55], with files excluded if more than 10% of the data required editing. Respiratory sinus arrhythmia was calculated from the edited IBI data using CardioBatchPlus V7 (Brain–Body Center for Psychophysiology and Bionegineering, University of North Carolina, Chapel Hill, NC, USA) [56] by applying a bandpass filter of 0.24–1.04 Hz and transforming variance into its natural logarithm. The mean IBI and RSA values were derived for use as dependent variables in this study. Descriptives are included in Table 2.

### 4.3. CGG Repeat Length

The diagnosis of the FXpm and genetic data regarding the length of each individual’s CGG expansion were both obtained through genetic analysis of the *FMR1* gene. The information was shared with this lab either through parental consent or through participation in the IBR SCL. Participants who had undergone prenatal screening at the IBR SCL had their FXpm diagnosis confirmed with a prenatal clinical report, which was shared upon parental authorization. To quantify the length of the CGG repeats, *FMR1* CGG repeat-primed PCR was performed with genomic DNA using the AmplideX^®^ PCR/CE *FMR1* Kit [57] as previously described [58,59,60]. For females, the higher CGG repeat length was used in analysis. Descriptives are included in Table 2.

### 4.4. Sensory Processing

The Sensory Experiences Questionnaire (SEQ; Version 2.1) [61] is a 43-item caregiver-report measure designed to assess children’s behavioral responses to sensory stimuli in everyday contexts for children aged 6 to 72 months. Behaviors are evaluated across both social contexts and non-social contexts and span multiple sensory domains, including auditory, tactile, visual, proprioceptive, gustatory, and olfactory systems. The SEQ assesses three primary patterns of sensory responsivity: hyporesponsiveness, hyperresponsiveness, and sensory seeking. In the present study, analyses focused on hyporesponsive and hyperresponsive subscale scores to examine sensory response patterns in infants with the FXpm and their NT peers. Items are summed to generate a subscale score, with higher scores reflecting greater frequency or intensity of sensory-related behaviors. The total score on the SEQ is categorized as normal, at-risk, or deficient according to the number of standard deviations above the mean score of the normative sample. Descriptives are included in Table 2. The SEQ has yielded acceptable internal consistency for children with neurodevelopmental conditions (Cronbach’s α: Hyperresponsiveness = 0.71, Hyporesponsiveness = 0.72, Total = 0.80) [61]. The SEQ has also demonstrated strong construct validity in children with ASD [61,62,63,64], FXS [25], and Angelman syndrome [65].

### 4.5. Procedures

Assessments took place in the participants’ homes or in a family-friendly research laboratory. Following parental consent, baseline cardiac activity was collected, followed by developmental assessments. Participants’ parents completed the SEQ as part of a series of questionnaires about their child’s development.

### 4.6. Statistical Analyses

Statistical analyses were performed using SPSS 27 [66] and R 4.5.3 [67]. Descriptive statistics were computed for all study variables and are reported as means and standard deviations and shown in Table 2. To assess whether groups differed in chronological age and biological sex, preliminary independent *t*-tests were conducted. Given the unequal sex distribution across groups, we tested sex differences for primary outcomes using independent samples *t*-tests; as sex was not associated with these measures, analyses were conducted on the full sample to preserve power. Potentially influential outliers were evaluated using Cook’s distance (threshold > 4/n) [68]; sensitivity analyses excluding influential cases did not change the pattern of results, so full-sample estimates are reported.

To address the first research question, differences in sensory processing between infants with the FXpm and their NT peers were examined. Independent *t*-tests were conducted to compare SEQ responsiveness scores between groups. Cohen’s *d* effect sizes were calculated for group comparisons and interpreted using standard conventions for effect size (i.e., 0.10 = small effect, 0.30 = medium effect, 0.50 = large effect) [69]. Fischer’s exact test was used to examine group differences in the proportion of infants categorized within the deficient or at-risk categories of the SEQ. Due to the low number of children in the deficient range, the deficient and at-risk categories were collapsed. To address the second research question, Pearson bivariate correlations were computed between baseline cardiac indices (i.e., RSA and IBI) and SEQ responsiveness scores for the NT and FXpm groups separately. A linear regression model predicting SEQ scores from CGG repeat length was conducted. To evaluate potential linear and non-linear associations reported in prior literature, both linear CGG and quadratic CGG^2^ were included as fixed effects. Statistical significance was evaluated at α = 0.05, and all reported *p*-values are two-tailed.

## Figures and Tables

**Table 1:** Sample demographic characteristics.

	FXpm (*n* = 35)	NT (*n* = 55)
Age (in months)		
Mean (*SD*)	11.34 (1.83)	11.29 (1.48)
Range	8.92–14.56	8.81–13.88
Sex		
Male	20 (57.14%)	40 (72.72%)
Female	15 (42.85%)	15 (27.27%)
Race		
White	30 (85.71%)	43 (78.18%)
Black/African Am.	0 (0%)	6 (10.91%)
Asian	0 (0%)	0 (0%)
More than one race	5 (14.28%)	6 (10.91%)
Ethnicity		
Hispanic	1 (2.86%)	4 (7.27%)
Non-Hispanic	34 (97.14%)	51 (92.73%)
MSEL ELC ^a^		
*N*	8	33
Mean (*SD*)	93.38 (12.37)	98.85 (13.87)
Range	78–118	71–123
BSID Cognitive SS ^b^		
*N*	27	22
Mean (*SD*)	94.26 (11.07)	94.95 (10.31)
Range	4–13	5–13

^a^ MSEL ELC = Mullen Scales of Early Learning Early Learning Composite Standard Score. ^b^ BSID Cognitive SS = Bayley Scales of Infant Development, 4th Edition, Cognitive Scale Standard Score.

**Table 2:** Descriptives for CGG repeat length, baseline cardiac measurements, and SEQ scores.

	FXpm (*n* = 35)		NT (*n* = 55)	
	*M* (*SD*)	Range	*M* (*SD*)	Range
CGG Repeat Length ^a^	71.85 (16.24)	55–117		
RSA	3.51 (1.05)	1.41–6.77	4.33 (1.14)	1.49–6.91
IBI	463.24 (40.40)	373.66–537.89	484.13 (48.09)	391.86–628.66
Hyporesponsive SEQ Score	1.48 (0.42)	1–2.5	1.45 (0.41)	1–2.67
Hyperresponsive SEQ Score	1.56 (0.31)	1–2.28	1.50 (0.36)	1–2.5
SEQ Category	*n* (%)		*n* (%)	
Deficient	1 (2.9%)		0 (0%)	
At risk	7 (20.2%)		9 (16.4%)	
Typical	27 (77.1%)		46 (83.6%)	

^a^ CGG Repeat available for 27 participants with the FXpm. For females, the higher CGG repeat length was used in analysis.

## Data Availability

The data presented in this study are available on request from the corresponding author due to privacy reasons given the sensitive nature of the study.

## Abbreviations

The following abbreviations are used in this manuscript:

ADHD	Attention-deficit/hyperactivity disorder
ANS	Autonomic nervous system
ASD	Autism spectrum disorder
BSID	Bayley Scales of Infant and Toddler Development
CGG	Cytosine–guanine–guanine
*FMR1*	Fragile X messenger ribonucleoprotein 1
FMRP	*FMR1* protein
FXAND	Fragile X-associated neuropsychiatric disorders
FXpm	Fragile X premutation
FXS	Fragile X syndrome
HRV	Heart rate variability
IBI	Interbeat interval
IBR SCL	Institute for Basic Research Specialty Clinical Laboratories
MSEL	Mullen Scales of Early Learning
NT	Neurotypical
RSA	Respiratory sinus arrhythmia
SEQ	Sensory Experiences Questionnaire
